# A Systematic Review of Herbal Interventions for the Management of Cardiovascular Diseases

**DOI:** 10.2174/011573403X286573240422104647

**Published:** 2024-05-03

**Authors:** Ankita Wal, Neha Verma, Senthil Kumar Balakrishnan, Vinod Gahlot, Sumeet Dwivedi, Pankaj Kumar Sahu, Mohammad Tabish, Pranay Wal

**Affiliations:** 1 PSIT-Pranveer Singh Institute of Technology (Pharmacy) NH19 Kanpur Agra Highway, Bhauti Kanpur, Uttar Pradesh, India;; 2 JKKMMRF'S Annai JKK Sampoorani Ammal College of Pharmacy, Komarapalayam, Tamilnadu, India;; 3 HIMT College of Pharmacy, Institutional Area, Knowledge Park - 1, Greater Noida- 201310, India;; 4 Acropolis Institute of Pharmaceutical Education and Research Indore, MP, India;; 5 Department of Botany, Govt. S.S.P. College Waraseoni, District Balaghat, India;; 6 Department of Pharmacology, College of Medicine, Shaqra University, Shaqra 11961, Saudi Arabia

**Keywords:** Heart attack, cardiovascular disease, anti-oxidant, cholesterol, herbs, lipids

## Abstract

**Background:**

Cardiovascular diseases represent a significant global health burden, necessitating diverse approaches for effective management. Herbal interventions have gained attention as potential adjuncts or alternatives to conventional therapies due to their perceived safety and therapeutic potential. This structured abstract provides a comprehensive review of herbal interventions for the management of CVDs, summarising key findings, mechanisms of action, and clinical implications.

**Objective:**

This systematic review aims to evaluate the impact of various herbal interventions employed for managing cardiovascular diseases.

**Methods:**

We conducted an extensive literature search across electronic databases, including PubMed, Scopus, and Web of Science, from inception to 2022. Studies were included if they investigated the use of herbal remedies for preventing or treating CVDs. Data extraction and synthesis focused on botanical sources, active compounds, mechanisms of action, and clinical outcomes.

**Results:**

Numerous herbal interventions have demonstrated promising cardiovascular benefits. A number of medicinal herbs well identified to treat CVD are *Moringaoleifera, Ginseng, Ginkgo biloba, Celosia argentea, Gongronematrifolium, Gynostemmapentaphyllum, Bombaxceiba, Gentianalutea, Allium sativum, Crataegus spp, Curcuma longa, Camellia sinensis, and Zingiber officinale.* Mechanistic insights reveal that herbal interventions often target multiple pathways involved in CVD pathogenesis. These mechanisms encompass anti-inflammatory, antioxidant, anti-thrombotic, anti-hypertensive, and lipid-lowering effects. Additionally, some herbs enhance endothelial function, promote nitric oxide production, and exert vasodilatory effects, contributing to improved cardiovascular health. Clinical studies have provided evidence of the efficacy of certain herbal interventions in reducing CVD risk factors and improving patient outcomes. However, more rigorous, large-scale clinical trials are needed to establish their long-term safety and effectiveness. It is crucial to consider potential herb-drug interactions and standardise dosages for reliable therapeutic outcomes.

**Conclusion:**

This comprehensive review highlights the potential of herbal interventions as valuable adjuncts or alternatives for managing cardiovascular diseases. Herbal remedies offer diverse mechanisms of action, targeting key CVD risk factors and pathways. While promising, their clinical utility warrants further investigation through well-designed trials to establish their safety and efficacy, paving the way for integrated approaches to cardiovascular disease management. Healthcare providers and patients should engage in informed discussions about the use of herbal interventions alongside conventional therapies in the context of CVD prevention and treatment.

## 1 INTRODUCTION

Cardiovascular diseases (CVDs) are conditions that negatively impact coronary blood vessels and the cardiac system [1]. Higher blood pressure has the highest level of probability for aetiology among CVD risk variables [2]. CVD encompasses a wide range of diseases, such as high blood pressure, lipid disorders, stroke, coronary heart disease, and heart failure.

It is the primary reason for deaths around the globe. CVDs killed an average of 17.9 million people in 2019, resulting in 32% of all worldwide fatalities. Heart attacks and strokes were responsible for 85% of these fatalities [3]. Ischemic heart disease and stroke were the two most prevalent forms of CVD health loss in each geographic location. It will kill more than 22.2 million individuals per year by 2030 [4, 5].

The Global Burden of Diseases, Injuries, and Risk Factors Study 2019, on which this publication is based, is a worldwide partnership that produces accurate and comparative health estimates from 1990 to 2019. It estimates the population health metrics for 204 nations and territories using all existing population-level information regarding occurrence, case fatality, mortality, and health risks [6].

According to the findings, global cardiovascular disease prevalence almost doubled from 271 million in 1990 to 523 million in 2019, while cardiovascular disease fatalities progressively grew from 12.1 million in 1990 to 18.6 million in 2019. Since 1990, nearly all cardiovascular disease fatalities worldwide have been caused by ischemic heart disease and stroke. In 2019, cardiovascular disease was the root cause of 9.6 million male fatalities and 8.9 million female deaths, accounting for around one-third of all deaths worldwide. Over 6 million of these fatalities occurred among people aged 30 to 70. China had the largest number of cardiovascular disease deaths, followed by India, Russia, the United States, and Indonesia [7, 8].

CVD is a worldwide health issue that necessitates a global strategy for prevention. It has the greatest impact in underdeveloped nations due to a lack of both financial resources and experts with competence in CVD prevention and management [9]. Conventional drugs widely used for managing CVD have negative effects as well as being prohibitively costly [10]. Hence, a safer, less expensive, and more powerful substitute is necessary. The most significant therapeutic alternative for cardiovascular illness is medicinal plants [11]. Herbal medicine has received considerable medical acceptance as a result of increased understanding of how herbs improve health. Growing evidence shows that consuming phytochemicals is an alternate and viable method for preventing CVD [12]. A range of cardiovascular illnesses have been treated with herbal medicines [13].

Naturally occurring compounds have been proven in recent research to successfully prevent, regulate, or inhibit major variables such as oxidative stress and inflammatory agents [13, 14]. Numerous plant bioactive substances, such as carotenoids, tocotrienols, diosgenin, isoflavones, and flavonoids, have been linked to a reduction in the risk of CVD. A randomised clinical trial on hypercholesterolemic patients found that consuming insoluble polyphenols might lower the cholesterol level, which is a marker of CVD risk [15].

The current study shows the relative significance of herbs that have therapeutic potential for controlling cardiac function and treating diseases associated with them. This might improve methods for finding novel drugs and aid in their development.

## 2 STUDY RATIONALE

This study aimed to investigate the rationality and efficacy of distinct herbal interventions used in the treatment of various cardiovascular diseases. Through a systematic review of the existing literature, this study sought to provide evidence-based insights into their potential impact on cardiovascular health. In addition, there is a short discussion of the clinical and preclinical studies included in this article.

## 3 METHOD

### 3.1 Study Design

**Fig. (1):**
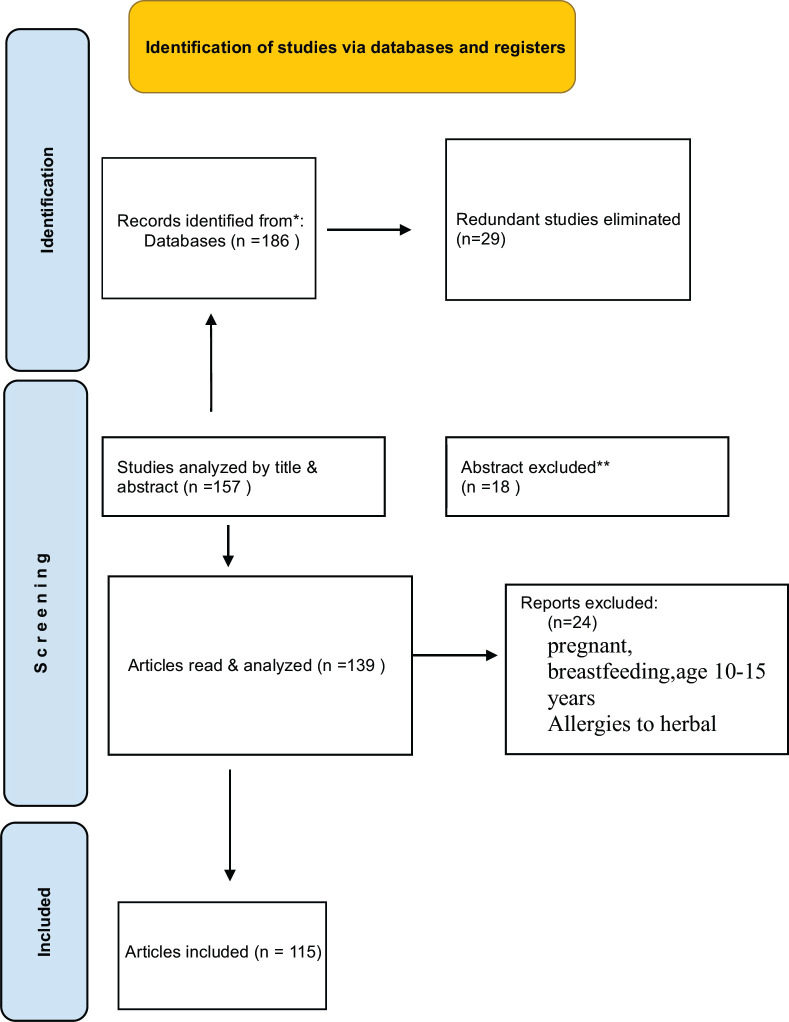
PRISMA flow diagram.

Following the standards outlined in Preferred Reporting Items for Systematic Reviews and Meta-Analyses (PRISMA), this systematic review was conducted without respect to time restrictions, allowing for a thorough analysis. There were no constraints on the language, publication year, or study design. Google Scholar and PubMed searches were used to find the data for this study. We looked through a wide range of research and review publications from several publishers, including ACS Publishing Centre, Springer Nature, Bentham Science, PLOS One, and MDPI, using keywords related to heart attack, cardiovascular disease, antioxidants, cholesterol, and Herbsto to find relevant information (Fig. **1**).

### 3.2 Inclusion Criteria

- Male and female individuals have past evidence of cardiovascular disease.
- Randomised controlled trials (RCTs)
- Observational study
- Meta-analyses
- Animal Studies

### 3.3 Exclusion Criteria

- Women who are pregnant, breastfeeding, or have a pregnancy plan.
- Participants within 4 weeks with a record of major organ surgery (*e.g*., head, chest, abdomen) or susceptibility to haemorrhage.
- Allergies to herbal treatment components used in studies.
- Aged between 10 and 15 years.

This review is trying to ensure scientific reliability by including and excluding studies based on established criteria. The goal is to ensure that the study featured meets certain criteria, delivering useful and credible information on certain herbs for cardiovascular disease treatment. This detailed strategy aims to enhance the overall quality and reliability of the review's findings, allowing for a more complete and reliable assessment of the efficacy of different herbs in the treatment of cardiovascular events.

## 4 RESULTS AND DISCUSSIONS

### 4.1 Factors that Increase Risk for Cardiovascular Disease

**Fig. (2):**
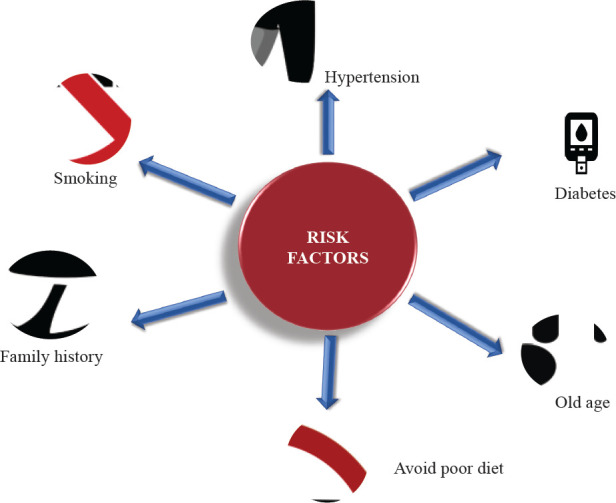
Risk factor of cardiovascular disease.

A variety of situations or events, such as inactivity, poor nutrition, or advanced age, can exacerbate CVD [16]. Factors are classified as modifiable or non-modifiable. Non-modifiable risk factors include a person's age, race, and family background (genetics cannot be modified). Modifiable risk factors such as smoking, nutrition, and exercise are a few examples (Fig. **2**) [16].

### 4.2 Family History

Patients under the age of 50 who have a previous family history of early heart illness are at a greater risk of coronary artery disease mortality [17]. This is typically the case if a person's first-degree relative had CVD while they were maybe still fairly young or if the person's parent had it before the age of 65 [18].

### 4.3 Smoking

Tobacco use is recognized as an important threatto CVD [19]. According to the European data, it is responsible for 30% of US CVD mortality. Not only is it harmful, but it is dosage dependent, with no safe lower limit seen. The most economical method of preventing CVD is quitting smoking, with some benefits beginning as soon as one month after quitting [20].

Animal models of nicotine exposure continue to exhibit CVD consequences, with high levels of atherosclerotic plaques identified in mouse models [21].

### 4.4 Poor Diet

Trans fat has been demonstrated through research to increase the chance of cardiovascular disease [22]. According to a 2016 systematic study, soft drinks were linked to a 22% high chance of myocardial infarction [23]. High intake of fructose, sucrose, and table sugar has all been implicated in the progression of coronary artery disease [24].

### 4.5 Hypertension

Hypertension is an independent major variable for the occurrence of CVD. The impact of raising blood pressure above 115/75 mmHg is constant and exponential, with each 20 mmHg rise in systolic blood pressure or 10 mmHg rise in diastolic blood pressure doubling the chance of heart problems [25].

### 4.6 Diabetes Mellitus

CVD is the major factor of death among diabetics [26]. When comparing adult sufferers with diabetes to those without diabetes, the chance of heart disease is 2.5 times higher in masculine and 2.4 times higher in ladies [27].

### 4.7 Socioeconomic Status

Cardiovascular disease appears to be more prevalent among those with low socioeconomic levels. Although the causes for this are numerous and their interrelationships complicated, food is often regarded as one of the most important, with individuals from higher socioeconomic backgrounds typically having more access to a more nutritionally balanced diet [28].

### 4.8 Plant used in the Management of CVD

Many studies have focused on using natural goods and herbal treatments to prevent or cure cardiovascular disease [29]. Particularly, the possibility for a financially viable course of therapy has been contrasted with the existing accepted standard of care and the widespread acceptance of its safety and efficacy.

In 1985, WHO reported that almost 65% of the global populace relied mostly on plant-based products [30]. Various clinical (Table **1**) and preclinical evaluations have proven the effectiveness of several herbs in the management of CVD (Table **2**).

#### 4.8.1 Ginkgo biloba

It is popularly named the maidenhair tree because its leaves resemble those of the maidenhair fern. It is considered a “living fossil” due to its 270-million-year life with no major alterations [31]. The beginning habitat is in China, Japan, and Korea and is planted across India, particularly in mountainous parts. The medicinal and pharmacological properties of Ginkgo biloba are mostly attributable to its component flavonoids and terpenoids [32]. Due to the well-known oxidation and inflammation-lowering properties of these Ginkgo biloba components, they are mostly utilized as a remedy for CVD

##### 4.8.1.1 Mechanism

Free radical production leads to the growth and progression of a variety of CVDs, such as vascular injury and plaque growth. In CVD, the balance between free radical formation and antioxidant defence shifts significantly. Its antioxidant effect serves to scavenge excess free radicals and limit free radical production, making it useful in the treatment of cardiovascular disease. *Ginkgo biloba* may also function as an antihypertrophic agent during heart hypertrophy by activating the M2 muscarinic receptor/NO pathway and cholinergic signalling [11].

#### 4.8.2 Panax ginseng

Ginseng refers to any of the perennial herbs of the Panax genus and Aralliaceae family. It is found in 35 nations, mostly in Northern America, primarily in colder regions, and in Eastern Asia, particularly in Japan, Korea, northeastern China, Bhutan, and East Siberia. Moreover, India, Nepal, and Myanmar are home to extremely important wild ginseng varieties with prolonged growth seasons [33]. Ginseng extracts have been shown to be anti-hyperglycaemic, reduce blood pressure, and insulin sensitivity, and have anti-hyperlipidaemic properties [34].

##### 4.8.2.1 Mechanism

The mechanism of action of *Panax ginseng* in cardiovascular disease involves multiple pathways and actions that contribute to its potential beneficial effects on the cardiovascular system. Some of the key mechanisms include:

a. ***Vasorelaxation***

Ginsenosides found in *Panax ginseng* promote the release of NO in the endothelial cells lining the blood vessels. Nitric oxide is a potent vasodilator, which means it relaxes and widens the blood vessels, leading to improved blood flow and reduced blood pressure. This effect can be beneficial for individuals with hypertension or other cardiovascular conditions [35].

b. ***Anti-hyperlipidemic***

Some research suggests that *Panax ginseng* can enhance lipid profiles by influencing lipid metabolism. Several mechanisms, including anti-inflammatory and anti-oxidative characteristics, have been postulated to explain ginseng's antihyperlipidemic actions in humans. These lipid-lowering effects may help to avoid cardiovascular disorders, including atherosclerosis [36].

#### 4.8.3 Glycine max

It is a member of the Fabaceae family and is a legume crop. Soybeans have been known to Indians for centuries since they were grown as a food plant in the northern and northeastern hills and are now a staple of the people's diet [37]. It is most commonly seen in China, the United States, Brazil, and Argentina. It contains a range of bioactive substances, including isoflavones, phytic acid, tocopherols, and saponins. These chemicals are related to a number of potential health advantages [38].

##### 4.8.3.1 Mechanism

The majority of the effect of trypsin inhibitors is removed by the heat-treatment process that is used on most soybean products [39]. Low concentrations of Bowman-Birk inhibitor were present that may have a hypocholesterolaemia impact *via* boosting cholecystokinin secretion. This would subsequently induce bile acid production from cholesterol, assisting in the elimination of cholesterol through the gastrointestinal system [40].

#### 4.8.4 Nigella sativa

It is a perennial flowering plant with green-to-blue blooms that can be grown and used across the world. It grows widely in Western Asia, the Middle East, and Eastern Europe. A variety of pharmacological activities were seen in the oil that was extracted from the seeds [41].

##### 4.8.4.1 Mechanism

It has been proven to improve lipid profiles by dropping LDL & triglycerides. At the same time, it may boost high-density lipoprotein (HDL) cholesterol, which is good for heart health [42].

The seeds of the plant are also high in antioxidants, which support the scavenging of harmful free radicals and the protection of cells from oxidative damage [1].

#### 4.8.5 Silymarin marianum

*Silymarin marianum*has been utilized over more than 2000 years to treat various diseases.The plant is native to Australia, Southern Europe, North Africa, North and South America, along with certain parts of Asia [43].

Silymarin is regarded as the most effective cardioprotective agent. It possesses antioxidant properties and protects from oxidative stress-induced, atherosclerosis, reduces blood pressure, and prevents cardiac damage [44].

##### 4.8.5.1 Mechanism

The antioxidant properties of silymarin help to reduce oxidative stress and swelling, which are the risk events for the progression of CVD [45].

#### 4.8.6 Moringa oleifera

It is typically found in the Sub-Himalayas, but it is currently being grown all over the world due to its medicinal properties. All species are indigenous to the Indian subcontinent, the Red Sea region, and areas of Africa, especially Madagascar. It may grow quickly in high-temperature areas and on terrain with little water availability [46]. It is not only high in nutrients, but it also includes anti-nutrients such as flavonoids, which prevent oxidation [47].

##### 4.8.6.1 Mechanism

It has been reported that *Moringa oleifera* inhibits apoptosis, enhances cardiac contractility, and protects the structural integrity of the heart. By reducing oxidative stress and inflammation, MO may also have cardioprotective effects [48]. It exhibits antioxidant and anti-inflammatory properties that are effective for hyperglycaemia, hypertension, dyslipidaemia, and obesity [49].

#### 4.8.7 Gynostemma pentaphyllum

*Gynostemma Pentaphyllum*is a perennial creeper that belongs to the Cucurbitaceae family. Jiaogulan is a common name for *G. pentaphyllum*, which may be cultivated in India, Nepal, Bangladesh & Japan [50, 51].

##### 4.8.7.1 Mechanism

*Gynostemma pentaphyllum* may exert its cardiovascular benefits through various mechanisms such as anti-oxidant by protecting blood vessels and heart tissues from damage caused by oxidative stress [52].

It may help reduce the amount of LDL cholesterol while increasing HDL cholesterol (the “good cholesterol”), promoting better cardiovascular health [53, 54].

#### 4.8.8 Gongronema latifolium

*Gongronema latifolium* Benth belongs to the Asclepiadaceae family. It is a nutritional/medicinal herb that is usually grown in rainforests in Nigeria and other tropical African countries [55].

It possesses diverse pharmacological properties such as hypolipidemic activity, preventing oxidation, reducing swelling, and tissue regenerative and restorative potentials, which are all crucial in cardiovascular disease management [56].

##### 4.8.8.1 Mechanism

The hypotensive activities of G. latifolium are facilitated by the synergistic action of the substances, most likely *via* the -adrenergic blocking pathway [57]. It also has anti-inflammatory properties, since it reduces inflammation in blood vessels and heart tissues [58].

#### 4.8.9 Celosia argentea

*Celosia argentea* belongs to the Amaranthaceae family, popularly called semen, it is a smooth annual plant that is commonly used as a leafy vegetable in Africa's rainforest region. It is also known as a troublesome weed in India and China. It grows best in full sun and should be planted in a well-drained region [59]. It has a variety of medicinal characteristics, including vasodilator, reduce swelling, anti-oxidant and hepatoprotective [60].

##### 4.8.9.1 Mechanism

It acts as a hypotensive agent. Its vasodilatory response is mostly due to its role in the nitric oxide/cGMP and prostaglandin/cAMP pathways. Furthermore, its actions are associated with the activation of voltage-dependent K^+^ channels [61].

#### 4.8.10 Citrus bergamia

*Citrus bergamia* is a member of the Rutaceae family, this fruit is mostly collected in the Calabria region of Southern Italy. Its peel oils are well-defined and widely utilized in goods ranging from the food sector to the pharmaceutical sector to the cosmetic industry. This fruit is most recognized for its antioxidant, lowering swelling, and cholesterol-lowering actions. Itrich in several bioactive compounds, such as flavonoids and polyphenols, that may contribute to its potential cardiovascular benefits [62].

##### 4.8.10.1 Mechanism

*Citrus bergamia* includes antioxidants such as flavonoids (naringin, neohesperidin, rutin) that help the body scavenge free radicals. Free radicals may induce oxidative stress, which damages blood vessels and has been demonstrated to improve levels of lipids by reducing LDL cholesterol, and triglycerides. These effects may contribute to a decreased risk of cardiovascular disease [62, 63].

#### 4.8.11 Gentiana lutea

It belongs to the family Gentianaceae and is indigenous to the mountainous regions of Central and Southern Europe, extending from Greece to Spain and extending as far as the northwest of Turkey. Additionally, it is dispersed throughout the hilly areas of China, the Vosges Mountains, Yugoslavia (formerly known as Croatia and Serbia), and Jura. Flavonoids, triterpenoids, and carbohydrates are abundant in Gentiana. The medicinal plants of the Gentiana genus have various activities like reducing swelling, preventing oxidation, reducing lipid concentration as well as preventing cardiac damage, improving blood flow, and hypotensive and anti-platelet action [64].

##### 4.8.11.1 Mechanism

It works through many mechanisms of blood pressure reduction caused by preventing Ca^2+^ entry and release from intracellular storage. it also prevents the buildup of plaque in the artery and cures by preventing the proliferation of vascular smooth muscle cells [65].

#### 4.8.12 Zingiber officinale

It is grown all over the world, including in regions of the United States, Australia, Brazil, China, India, Jamaica, and West Africa [66].

Ginger is a natural remedy that has been demonstrated to have powerful anti-diabetic, anti-inflammatory, anti-oxidant, anti-microbial, and other effects [67]. Its crude extract is employed as a cardioprotective drug due to its antihypertensive and antiplatelet effects [68].

##### 4.8.12.1 Mechanism

Ginger's antihypertensive properties are attributed to 6-shogaol and 9-gingerol. These substances lower cholesterol and LDL levels, limit the development of atheroma plaques, and promote vascular flexibility. They also inhibit the production of inflammatory mediators that cause endothelial dysfunction by reducing the levels of intercellular adhesion molecule 1 (ICAM-1) [69].

#### 4.8.13 Curcuma longa

The Curcuma genus, which includes about 120 species, has a long tradition of therapeutic usage. Turmeric (Curcuma longa) and other curcuma species grow in the forests of Southern Asia, especially India, Indonesia, and Indochina, as well as surrounding Asian nations and Pacific Islands such as Hawaii [70].

*Curcuma longa* L. is one of the most commonly known Curcuma species; a variety of beneficial medicinal properties have been obtained from the Curcuma species, including anti-inflammatory, anticancer, antidiabetic, hypocholesterolemic, anti-thrombotic, hypotensive, antimicrobial, antiviral and antioxidant, impacts, among others [71, 72].

##### 4.8.13.1 Mechanism

Curcumin has been found to have anti-arteriosclerotic properties by protecting against inflammation and oxidation, modulating cholesterol equilibrium, and inhibiting platelet aggregation. Curcumin has been shown in studies to reduce low-density lipoprotein while increasing high-density lipoprotein [73].

Different clinical and preclinical evaluations of several herbs on cardiovascular disease. As shown in Tables **1** and **2**.

## FUTURE DIRECTIONS

**Conducting high-quality RCTs:** Rigorous clinical trials with standardized herbal products are essential for confirming the efficacy and safety of promising herbal interventions for CVD. This includes investigating active ingredients, optimal dosages, and potential side effects.

**Standardization of herbal products:** Establishing quality control standards for herbal products, including active ingredient content, purity, and safety testing, is crucial for ensuring reliable therapeutic outcomes.

**Investigating mechanisms of action:** Understanding the specific mechanisms by which herbal interventions work can guide the development of targeted therapies and combination strategies with conventional medications.

**Addressing herb-drug interactions:** Thorough research on potential interactions between herbs and conventional medications is necessary to develop safe and effective treatment plans for patients with CVD.

**Patient education and informed consent:** Healthcare providers need to educate patients about the potential benefits and risks of herbal interventions for CVD, emphasizing the importance of informed consent and collaboration with healthcare professionals when exploring herbal therapies.

**Integrating herbal medicine into CVD management:** Future research should explore the potential for integrating evidence-based herbal interventions into comprehensive CVD management strategies alongside conventional therapies, with a focus on personalized medicine approaches.

By addressing these limitations and pursuing these future directions, herbal interventions can potentially play a valuable role in the prevention and treatment of cardiovascular diseases, offering patients additional options and complementing conventional therapies for improved cardiovascular health.

## CONCLUSION

In conclusion, the herbal management of cardiovascular disease has shown promising potential as a complementary approach to conventional treatments. Throughout history, various herbal remedies have been utilized for their purported cardiovascular benefits, and scientific research has started to shed light on their mechanisms of action and efficacy. Herbal medicines like *Curcuma longa, Moringa oleifera, Ginseng, Ginkgo biloba*, *Celosia argentea, Gongronema trifolium and Gynostemma pentaphyllum* have demonstrated positive effects on cholesterol levels, blood pressure regulation, anti-inflammatory properties, and antioxidant activities. These properties are crucial in managing and preventing cardiovascular diseases, including hypertension, atherosclerosis, and heart disease.

More research is needed to validate the toxicity and long-term effectiveness of herbal remedies for cardiovascular diseases. Collaborative efforts among researchers, healthcare providers, and herbal practitioners can help integrate these treatments into mainstream healthcare, ensuring a comprehensive and holistic approach to cardiovascular disease management.

## LIMITATION

While many studies suggest the potential benefits of herbal interventions for CVD, many lack the rigor of large-scale, randomized controlled trials (RCTs). These are crucial for establishing true efficacy and safety compared to conventional therapies. Although composition and potency of herbal remedies can differ significantly due to factors like growing conditions, processing methods, and storage. Standardization of herbal products is essential for ensuring reproducible results in clinical trials and clinical practice.

Potential interactions between herbs and conventional medications pose a safety concern. More research is needed to identify and understand these interactions for safe and effective co-administration. The long-term safety and efficacy of many herbal interventions for CVD are largely unknown. Long-term follow-up studies are needed to assess potential risks and benefits over time.

## Figures and Tables

**Table 1:** Clinical evaluation of several herbs on cardiovascular disease.

**Herb**	**Formulation**	**Duration**	**Study type**	**Result**	**Safety**	**References**
*Curcuma longa*	1. Curcumin extract (low dosage 3 times 15 mg/day; medium dose 3 times 30 mg/day; large dose 3 times 60 mg/day)	10 months	Randomized double blind controlled trial performed on 72 acute coronary syndrome patients.	Lowered the level of total and LDL cholesterol.	Safe, well-tolerated and efficacious	[74]
	2. Curcuminoids supplement (1000 mg/day co-administered with piperine 10 mg/day)	8 weeks	Randomized placebo-controlled trial performed on 118 subjects.	Curcumin treatment lowered the chance of developing acute cardiovascular events in patients suffering from type 2 diabetes along with dyslipidemia	No significant adverse effects.	[75]
	3. Curcuminoids supplement (1 g/day)	1 month	Randomized Crossover Trial performed on 30 patients.	Curcuminoid administration leads to a considerable decrease in blood triglyceride levels.	No significant adverse effects.	[76]
*Gentiana lutea*	1. Extract of bergamot (150 mg/day)	6 months	open-label clinical trial performed on 80 human subjects (42 men and 38 women,) with moderate hypercholesterolemia.	Bergamot was observed to decrease total cholesterol (LDL and triglycerides). An increase in HDL level	Not available	[62]
	2. Two capsules each day (total 500 mg of bergamot fruit extract & 220 mg of phytochemical complex combination).	12 weeks	An observational, one-arm research was done with 11 human subjects to assess a mixture of 9 plant extracts, including bergamot fruit extract.	A decrease in the levels of total cholesterol, LDL cholesterol, and apolipoprotein B was identified.	safe and effective	[77]
	3. 500 mg /day Bergamot Polyphenol Fraction (BPF) 1000 mg/day (BPF) Placebo tablet had no active ingredients	1 month	Randomized, double blind, placebo-controlled performed on 237 subjects.	Lowered the level of total and LDL cholesterol as well as increased HDL level	Mild stomach pyrosis was found.	[78]
*Ginkgo biloba*	1. *Ginkgo biloba* extract (300 mg/day)	4 month	A double-blind, placebo-controlled trial was performed on treadmill walking time among 62 adults with peripheral artery disease.	Ginkgo biloba generated a minor increase in maximum treadmill walking duration and flow-mediated vasodilation in older persons with peripheral artery disease.	No significant adverse effects.	[79]
	2. *Ginkgo biloba* pills and placebo	58 weeks	Randomized, double-blind, placebo-controlled, three-period crossover trial performed on 12 subjects.	Improving glucose regulation can prevent diabetes and lower the risk of cardiovascular disease.	Not available	[80]
	3.*Ginkgo biloba* dropping pills	12 weeks	Randomised, double-blind, placebo controlled, parallel-group and multicentre clinical trial conducted in 72 patient	It improves the frequency and quality of life for people with stable angina pectoris and depressive symptoms.	Dizziness, fatigue, facial flushing and dry throat	[81]
*Zingiber officinale*	1. Ginger capsules 3 g/day in 3 split doses, and the control group received lactose capsules 3 g/day in 3 split doses	45 days	A double-blind-controlled trial was performed on 45 patients	Ginger significantly reduces the low-density lipoprotein, triglycerides, and cholesterol	Not available	[82]
	2. Ginger supplement (3 g/day)	30 days	Meta-analysis of prospective studies performed on 100 Hyperlipidemic patients	Significantly reduce triglyceride	Not available	[83]
	3. Ginger powder in capsules (3 g/day)	8 weeks	Randomized, double-blind, placebo-controlled trial on 88 sufferers	Significantly reduce LDL level.	Not available	[84]

**Table 2:** Preclinical evaluation of several herbs on cardiovascular disease.

Study	Methodology	Result	References
To investigate Ginseng's potential to prevent established cardiomyocyte and heart failure	Ginseng was given to rats in drinking water after 4 weeks of prolonged coronary artery ligation when heart failure developed	This study shows that ginseng has a significant ability to reverse cardiac hypertrophy, myocardial remodeling, and heart failure,	[85]
To investigate *Ginkgo biloba* extract enhances cardioprotection in an isoproterenol-induced heart hypertrophic model.	Male Wistar rats were separated into four experimental groups and treated for eight days: 1: The control group was given saline. 2^nd^ was given isoproterenol, 3^rd^ was given *Ginkgo biloba* extract. 4^th^ received isoproterenol with *Ginkgo biloba* extract.	*Ginkgo biloba* proved effective in decreasing the adverse cardiac events brought on by cardiac hypertrophy.	[86]
The anti-hyper lipidemic impact of crude methanolic extracts of *Glycine Max* on high cholesterol diet-fed albino rats.	For two weeks, male rats were fed a high-cholesterol diet and *Glycine max* at doses of 400 mg/kg and 200 mg/kg, respectively, as well as atorvastatin 20 mg/kg.	The glycine-consuming group was found to have an anti-hyper lipidemic impact on hyperlipidemic rats and to have cholesterol-lowering property	[87]
To examine the preventive effects of *Nigella sativa* on cadmium cardiotoxicity in albino rats.	Male albino rats were divided into five groups, with one serving as a control and the others receiving varying doses of cadmium chloride for seven days.	It has been shown that *Nigella sativa* has cardioprotective effects, from harmful substances like cadmium	[88]
To examine Cardioprotective action of silymarin in myocardial infarction in albino rats	Wistar albino rats were taken and silymarin was given in three distinct dosages for one week.	Silymarin has cardioprotective action in rats because it inhibits neutrophil infiltration during ischemia-reperfusion and protects endogenous antioxidant enzymes.	[89]
Cardioprotective Activity of *Moringa oleifera* on Doxorubicin-Induced Cardiotoxicity in Rats.	female Sprague Dawley rats were separated into five groups and administered doxorubicin with *Moringa oleifera* Leaf Ethanolic Extract orally for two weeks.	The results demonstrate that *Moringa oleifera* is effective in preventing cardiotoxicity	[90]
Cardiovascular effects of the aqueous extract of *Gynostemma pentaphyllum* in guinea-pigs.	*G. pentaphyllum* decoction was administered to guinea pigs at doses of 2.5, 5, and 10 mg/kg.	Protective effect against pitressin-induced coronary spasms, arrhythmias, *etc.*, indicating that it functions as a cardioprotective agent	[91]
*Gongronema latifolium* (GL) leaf extract protects against dexamethasone-induced myocardial cell injury	Wistar rats were given dexamethasone and dexamethasone plus GL extract subcutaneously to cause myocardial damage, and GL leaf extract orally for 14 days.	Extract has anti-inflammatory and cardioprotective activities as well as an effective free radical blocker with high antioxidant capacity.	[58]
To examine the *Celosia argentea* L. a vasodilator plants.	Three different dosages of Celosia argentea (30, 100, and 300 mg/kg) were tested for hypotensive effects in male Wistar rats.	The findings demonstrated a Hypotensive effect of *Celosia argentea* in normotensive rats.	[61]
To investigate the *Gentiana lutea* has anti-atherosclerotic properties in streptozotocin (STZ)-induced diabetic rats.	STZ rats were given 2% Gentiana lutea root powder orally as a supplement to their usual diet for 30 days.	The findings demonstrated that *Gentiana lutea* root powder/extract has anti-atherosclerotic properties.	[92]
Hypolipidemic Action of *Citrus bergamia* Juice in wistar rats	Three groups of Wistar rats were formed as follows: control rats with normal cholesterol levels, hypercholesterolemic rats, and hypercholesterolemic rats taking bergamot juice for 30 days.	Results indicate that consumption of *C. bergamia* may lower the risk of several heart diseases due to its activity as a radical scavenger and hypocholesterolemic properties.	[93]

## Data Availability

The authors confirm that the data supporting the findings of this research are available within the article.

## LIST OF ABBREVIATIONS

CVDs: Cardiovascular Diseases
RCTs: Randomised Controlled Trials
HDL: High-Density Lipoprotein

## STANDARDS OF REPORTING

PRISMA guidelines and methodology were followed.

## SUPPLEMENTARY MATERIAL

PRISMA checklist is available as supplementary material on the publisher’s website along with the published article.
